# Analyzing Social Media Trends in Cosmeceuticals: Insights From Google Trends and TikTok Analytics

**DOI:** 10.1111/jocd.70172

**Published:** 2025-04-08

**Authors:** Emily Correia, Jenna Mandel, Stephanie R. Jackson Cullison

**Affiliations:** ^1^ Department of Dermatology and Cutaneous Biology Thomas Jefferson University Philadelphia Pennsylvania USA

**Keywords:** cosmeceuticals, Google Trends, niacinamide, retinol, search trends, social media

## Abstract

**Objective:**

Social media usage has surged, particularly since the COVID‐19 pandemic. Dermatology invokes intense interest on social media, and cosmeceuticals are among the most trending topics, offering a unique space for dermatologists to share their expertise. By using tools like Google Trends and TikTok viewership, we aim to capture cosmeceutical trends to guide dermatologists' educational efforts.

**Methods:**

A Google Trends search from January 2004 to December 2023 was performed on retinol, bakuchiol, salicylic acid, glycolic acid, azelaic acid, hydroquinone, niacinamide, vitamin C serum, and hyaluronic acid. Trend lines were created for each term, and yearly percent differences in relative search volume index (SVI) were calculated. Relative interest for each cosmeceutical was analyzed, comparing the average SVI and TikTok views.

**Results:**

Google data shows sustained cosmeceutical search volume growth, with the largest increase from 2020 to 2021, coinciding with the COVID‐19 pandemic. Cosmeceutical search volume corresponds with TikTok views. Both platforms demonstrate retinol as the most popular cosmeceutical, followed by hyaluronic acid, salicylic acid, glycolic acid, and vitamin C. Niacinamide has considerably more TikTok views compared to search interest. Bakuchiol was the least popular cosmeceutical.

**Conclusions:**

The results reveal growing curiosity in cosmeceuticals, with interest correlating with consumers' views on social media. Awareness of skincare trends and growth in cosmeceutical interest empower dermatologists to anticipate patient inquiries and develop targeted education on product efficacy, cost effectiveness, and potential adverse reactions. Social media platforms are a possible space for dermatologists to engage with their patients and ensure dissemination of accurate cosmeceutical information.

## 1 Introduction

The surge in social media usage over the past decade has inspired a growing interest in Dermatology among online communities. This trend was amplified during the COVID‐19 pandemic, as school and business closures encouraged reliance on virtual platforms. Consequently, online social networks became convenient, free sources of information for individuals with dermatologic concerns. Increased use of video technology may have accentuated perceived facial imperfections as individuals viewed themselves more frequently on screens. Promotion of tools available to improve appearance on digital platforms (filters, lighting) likely drew attention to the role of external perceptions in professional advancement. Finally, additional time at home may have intensified self‐criticism, further driving interest in aesthetics [1, 2]. In this digital age, cosmeceuticals—cosmetics designed to produce physiologic effects on skin—gained significant popularity [3].

Surveys suggest a substantial portion of adults (45%–50%) consult social media for skin care advice [4, 5]. However, a recent analysis of dermatology‐related Instagram hashtags found that only 4% of highly trafficked social media accounts disseminating dermatology‐related content are managed by board‐certified dermatologists [6]. Although 38% of content creators in dermatology claim to be healthcare professionals, the majority fail to disclose credentials, raising concerns about the accuracy of content [6, 7]. Nearly 45% of dermatology information portrayed on social media has been deemed imprecise, and approximately 66% of disease‐specific content on platforms like YouTube has been characterized as dangerous or misleading [8, 9]. Consequently, there is a need for dermatologists to disseminate accurate information about skincare and address social media misinformation to protect consumers.

Dermatologists may have multiple reasons for avoiding content creation on social media, including time, liability or privacy concerns, and technology naivety. However, insufficient education and training may also present a barrier to content creation. Studies show dermatology residents feel inadequately prepared to engage in educational discussions regarding cosmeceuticals for skin care [10]. Leveraging trending dermatology content on social media platforms may allow us to identify areas for educating dermatologists and trainees and creating content relevant to patients.

Google Trends is an online database and analytic platform measuring the relative popularity of search terms within a given timeframe, providing real‐time information on population interests [11, 12]. Various studies using Google Trends have found that dermatology is the highest‐searched medical specialty. Additionally, the high volume of dermatology‐related Google searches correlates with the proportion of self‐referred patients, suggesting that online searches may influence patients' decisions to seek dermatologic care. Prior Google Trends analyses have been used to evaluate the popularity of searches related to skin cancers, pruritus, cosmetic procedures, atopic dermatitis, and psoriasis [12]. Additionally, other reports have investigated TikTok content, rating the accuracy of discussions on hyperhidrosis, keratosis pilaris, and onychomycosis [13, 14, 15, 16]. Herein, we report the first Google Trends analysis evaluating searches for common cosmeceutical agents and correlate these findings with viewership of related content on TikTok.

## 2 Materials and Methods

Google Trends is a public tool that calculates search term prevalence relative to the total number of searches on the platform. The search volume index (SVI) represents the ratio of the frequency a term is searched within a specific time relative to its peak search volume within that timeframe. Google Trends therefore can be used to identify the popularity of a search term over time. An SVI of 100 represents the peak popularity for the search term, and any other value indicates a percentage of the total peak interest at any given time.

In February 2024, a Google Trends search was performed for multiple cosmeceuticals including retinol, bakuchiol, salicylic acid, glycolic acid, azelaic acid, hydroquinone, niacinamide, vitamin C serum, and hyaluronic acid. These specific cosmeceuticals were chosen as they had the most data on the Google Trends database. The search was limited to the United States from January 2004 to December 2023. Using data generated from Google Trends, scatterplots were created for each search term with fitted trend lines. The horizontal axis represented time and the vertical axis conveyed the SVI of the cosmeceutical. Percent differences in average SVI (search interest) between each year were calculated to determine the increase or decrease in popularity of the search term.

Search trends comparing the popularity of cosmeceuticals were graphed on scatterplots with fitted trend lines. Average search interest was compared to views on TikTok. To determine TikTok views, each cosmeceutical was input into the search function on TikTok, and the number of views for each term was obtained. TikTok was selected over other social media sites such as Instagram or YouTube because its viewership and search data is publicly accessible via its interface, and published studies support its role as an important tool for dissemination and digestion of unregulated health information by consumers. Trend analyses were performed using Microsoft Excel Version 16.60 (Microsoft, Redmond, WA, USA).

## 3 Results

**FIGURE 1:**
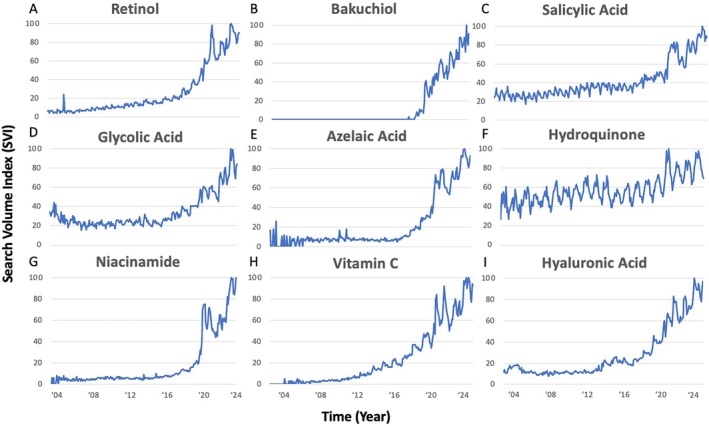
Google search volume interest from January 2004 to December 2023 for retinol, bakuchiol, salicylic acid, glycolic acid, azelaic acid, hydroquinone, niacinamide, vitamin C, and hyaluronic acid was plotted over time in years. SVI represents the ratio of the frequency a term is searched within a specific time relative to its peak search volume within that timeframe, such that an SVI of 100 is considered the peak search volume.

Google Trends demonstrates search interest for cosmeceuticals is constantly increasing, particularly after COVID‐19 shutdowns. Trend analysis shows the largest increase in search interest for all cosmeceuticals from 2020 to 2021, correlating with the shutdowns (Table 1). The average SVI of cosmeceuticals doubled or tripled in 2020, with some increasing by over 40%. However, search interest dipped the following year (2021 to 2022), likely when people returned to work, with some cosmeceuticals seeing up to a 14% drop in interest. Nevertheless, search interest surged again the subsequent year (2022 to 2023) demonstrating its second‐highest increase. Overall trends reveal a continuous search interest increase over time, indicating a growing curiosity with cosmeceuticals (Table 1, Figure 1).

**TABLE 1:** Average search volume index (SVI) differences per year for cosmeceuticals from 2018 to 2023.

Cosmeceutical	Average SVI differences by year					
	2018	2019	2020	2021	2022	2023
Retinol	+ 7%	+16%	+36%	−7%	+18%	−9%
Bakuchiol	+8	+16%	+24%	11%	+17%	+4%
Salicylic acid	+10%	+2%	+25%	−9%	+20%	+2%
Glycolic acid	+4%	+17%	+1%	−2%	+15%	+12%
Azelaic acid	+9%	+11%	+41%	−11%	+26%	−7%
Hydroquinone	+4%	+1%	+11%	+2%	+9%	−11%
Niacinamide	+2%	+14%	+42%	−14%	+25%	+18%
Vitamin C	+4%	+7%	+33%	−7%	+23%	−4%
Hyaluronic acid	+14%	+13%	+24%	−3%	+12%	+5%

*Note:* The percentage increase (indicated by +) or decrease (indicated by −), based on average Google SVI was calculated per year. SVI represents the ratio of the frequency a term is searched within a specific time relative to its peak search volume within that timeframe. Each column shows the SVI differences as a percentage from January 1st of the year listed until January 1st of the following year. COVID‐19 shutdowns notably began in March 2020.

**FIGURE 2:**
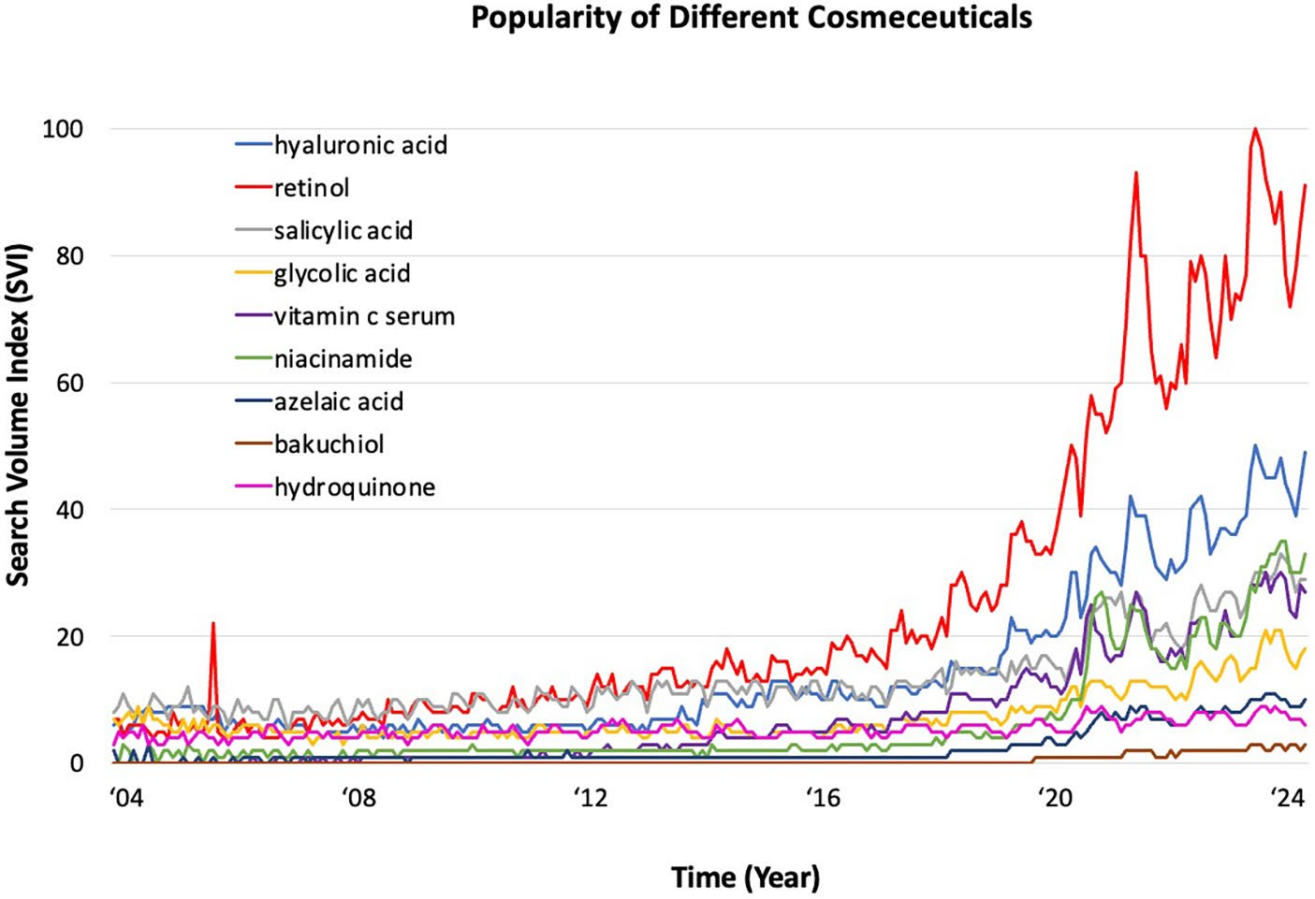
Popularity comparison for common cosmeceuticals based on Google search volume index (SVI) over time in years. SVI represents the ratio of the frequency a term is searched within a specific time relative to its peak search volume within that timeframe, such that an SVI of 100 is considered the peak search volume.

Scatterplots comparing SVI over time for different cosmeceuticals allowed analysis of the relative popularity of each cosmeceutical (Figure 2). Average search interest for each cosmeceutical was examined to further characterize the popularity of search terms. Retinol demonstrated the most interest, and Bakuchiol showed the least interest by Google Trends SVI (Table 2). Comparison of SVI and the number of TikTok views for each cosmeceutical demonstrated retinol as the most popular cosmeceutical across both platforms. Google search volume and TikTok views for other cosmeceuticals do not correspond directly, but a correlation appears to exist. For example, hyaluronic acid, salicylic acid, niacinamide, and vitamin C have high search volumes while having billions of TikTok views, whereas hydroquinone, azelaic acid, and bakuchiol have both lower search volumes and TikTok views. Niacinamide notably has a larger number of TikTok views relative to its Google SVI, whereas salicylic acid has a relatively higher Google SVI versus its TikTok views. Although not perfectly aligned, these data results collectively reveal that cosmeceutical appeal is constantly increasing, and that interest correlates with consumer viewership on social media.

**TABLE 2:** Average cosmeceutical search volume index (SVI) and corresponding TikTok viewership.

Cosmeceutical	Average SVI on Google	Number of TikTok views (million)	Interest rank by Google SVI	Interest rank by TikTok views
Retinol	25.01	8700	1	1
Hyaluronic acid	14.51	3200	2	3
Salicylic acid	13.49	1300	3	6
Glycolic acid	7.25	2100	4	4
Vitamin C	7.23	1400	5	5
Niacinamide	6.38	4900	6	2
Hydroquinone	5.35	237.8	7	9
Azelaic acid	2.47	357.4	8	7
Bakuchiol	0.42	351.9	9	8

*Note:* This table depicts the average SVI based on the relative number of Google queries, and the total TikTok views for each listed cosmeceutical from 2004 to 2023. SVI represents the ratio of the frequency a term is searched within a specific time relative to its peak search volume within that timeframe. Interest rank (highest to lowest average SVI or viewership) is also shown for each cosmeceutical to facilitate direct comparison across platforms.

## 4 Discussion

Our findings reveal a twofold increase in online queries for cosmeceutical products during the COVID‐19 pandemic, with a sustained upward trend. This surge in Google queries reflects growing interest in skincare and the influence of social media‐driven dermatology content. Between 2022 and 2023, search volume for the cosmeceuticals in this report rose by over 18% and is expected to continue increasing alongside social media engagement. For instance, TikTok is currently the fifth most‐used social media platform with over 1.5 million monthly users and is projected to reach 2.2 billion users by 2027 [17, 18].

While tools measuring viewership and searches cannot directly link trends to their underlying drivers, they likely reflect a complex interplay of cosmeceutical relevance (usage and consumer demand), formulation variety, accessibility (cost, regulation, safety), influencer or marketing efforts, and broader social or political factors. For example, hydroquinone's popularity has steadily increased, whereas bakuchiol and azelaic acid spiked in 2020. This surge coincides with the pandemic‐driven rise in cosmeceutical interest, but also aligns with the Coronavirus Aid, Relief, and Economic Security (CARES) Act's mandate to replace hydroquinone with other lightening agents in over‐the‐counter formulations [19]. Associated product access or marketing changes may have increased search and viewership for non‐hydroquinone lightening agents. Similarly, concerns over sunscreen and benzoyl peroxide safety grew following reports of high benzene levels in certain products [20]. Recognizing these influences can help dermatologists anticipate consumer questions and shape effective educational content on social media.

Despite the widespread popularity of cosmeceuticals and general dermatologist consensus on their potential skin benefits, evidence for cosmeceutical efficacy remains limited. Unlike pharmaceuticals, cosmeceuticals are not subject to pre‐approval for marking or distribution, nor are they required to demonstrate efficacy [21]. Aggressive marketing often promotes these products despite a lack of standardized evaluation, and no head‐to‐head comparisons exist to determine whether certain formulations outperform others. Ingredient concentrations vary widely, with labeling inconsistencies further complicating consumer interpretation. Few products such as niacinamide, vitamin C, salicylic acid, and hyaluronic acid have supporting data for efficacy at over‐the‐counter concentrations. For example, while 15% azelaic acid improves rosacea and acne, most over‐the‐counter formulations contain less than 15% and lack clinical studies supporting efficacy [22]. Over‐the‐counter salicylic acid cleansers with 2% concentrations are proven safe and effective for acne vulgaris [23]. Glycolic acid, commonly in many over‐the‐counter topicals, is deemed effective for anti‐aging effects in a concentration‐dependent manner [24], but increasing concentrations also increase the risks of adverse effects. Retinol has strong evidence supporting its ability to reduce wrinkles, pigmentation, elasticity, and photodamage, though it requires a minimum concentration of 0.025% [20, 25]. Bakuchiol, a retinol alternative, reportedly offers similar anti‐aging effects at concentrations of 0.5% as 0.05% tretinoin, though the literature describing Bakuchiol is limited [26, 27]. Hydroquinone effectively treats hyperpigmentation; however, it can cause irritation, sensitization, and ochronosis It is no longer available over the counter in the U.S. [28]. As a result, it is not commonly used in cosmeceutical products, which may explain the infrequent online searches. Additionally, no direct comparisons assess the efficacy of affordable versus high‐end products. Given that consumers may not recognize bias in product promotion on social media or may assume any cosmeceutical concentration guarantees results, dermatologists play a crucial role in guiding patients toward evidence‐based skincare choices.

Recently, the American Society for Dermatology Surgery (ASDS) released results of its Consumer Survey of Cosmetic Dermatologic Procedures, which suggested that dermatologists are the leading resource for skincare products and the primary influencer in consumer decisions. Social media ranked second in influencing skincare purchases [25]. A small number of board‐certified dermatologists are creating content, and organizations such as the American Academy of Dermatology (AAD), the American Society of Dermatologic Surgery (ASDS) and Skin of Color Society (SOCS) are building a social media presence. Nevertheless, very few board‐certified dermatologists and dermatology practices have a professional social media presence, and skincare advice on social media is still mostly provided by non‐dermatologists and paid content creators [6, 29].

Our data underscore the public's strong interest in skincare, a field where dermatologists are experts. Staying informed on popular social media trends enables dermatologists to engage in educational discussions on cosmeceutical efficacy and usage, helping patients identify suitable, cost‐effective products. The ingredients identified in this study may aid dermatology educators in addressing knowledge gaps and enhancing confidence in addressing cosmeceuticals with patients [10]. The American Association of Cosmetic Dermatology (AACD) is also developing educational content on cosmeceuticals and other aesthetic topics that may serve as valuable resources. This research highlights popular content that dermatologists can leverage to expand their social media presence and counter inaccurate or misleading content.

For dermatologists interested in creating engaging and educational social media content, the AAD offers social media resources including the “Your Dermatologist Knows” campaign, which partners with dermatologists to address trends and debunk myths. Collaborating with professional organizations can help standardize and amplify accurate dermatologic content and physicians should align their efforts with these organizations whenever possible. Several other online resources have tools for creating engaging and educational social media content. A general approach is to identify target audience(s) and post consistently, utilizing more interactive (video) versus passive (photo or text only) posts that entertain, inspire, or shed additional light on trending topics for content consumers while maintaining a personable, yet professional tone. Audience targeting is key, with patients likely interested in disease, procedure, or product‐specific education, and residents or medical students interested in dermatology and mentorship or career advice. Trending topics or disseminating high‐impact journal content can also yield very successful content. One of the unique challenges for physician content creators is the consideration of ethical and legal standards. Factors such as preserving patient privacy, refraining from individualized medical advice, maintaining regulatory compliance, and focusing on best practices and evidence‐based recommendations is essential to prevent or dispel misinformation.

While some dermatologists may prefer not to maintain a public social media profile, dermatology practices and professional organizations can use existing social media campaigns to share consensus statements or validated resources, such as those published by the AAD, and promote accurate skincare information. They may use this research to encourage broader social media presence within institutions, practices, or advocacy groups [30].

There are some limitations of Google Trends and TikTok in accurately assessing public interest in cosmeceuticals. Lower socioeconomic status and educational backgrounds may limit access to computers or smart devices needed for these search tools. Additionally, older patients may not be precisely captured in these analyses, as they may not interface with technology, including the latest applications, in the same way as younger generations. Finally, there may be regional variation in trends that are not captured in the current analysis. While Google Trends data can be localized to certain countries or geographic regions, TikTok searches cannot. Thus, the ability to directly compare Google and TikTok search trends is limited, as Google Trends data focuses on the US while TikTok data is international. Furthermore, searches for cosmeceutical ingredients that are also used in oral formulations for non‐cosmetic indications, such as niacinamide supplementation, may skew the popularity of these agents in our study.

Future studies could involve identifying barriers to social media presence among dermatologists or advocating for standardized disclosure of credentials and conflicts of interest for content creators. For example, the AAD YouTube Channel was recognized by YouTube as a credible source for health information in 2023, which gives it prominence in searches and has a label indicated it is a trustworthy source. Furthermore, evaluation of the engagement of specific demographics could shed light on their unique contributions to shaping trends in dermatology and guide more personalized content creation. Efforts such as the AAD “Your Dermatologist Knows” campaign have already started to use demographic targeting, with most of its content across a variety of social media platforms specifically directed toward women aged 25–35. Finally, as the utilization and capabilities of artificial intelligence (AI) continue to advance, it may be valuable to leverage AI tools to analyze modern data and predict future cosmeceutical trends.

## Author Contributions

**Emily Correia:** conceived the idea, designed the study, collected data, performed statistical analysis, and contributed to the final version of the manuscript. **Jenna Mandel:** helped perform statistical analysis and contributed to the final version of the manuscript. **Stephanie R. Jackson Cullison:** designed the study, performed analysis and interpretation of results, and contributed to the final version of the manuscript. All authors provided critical feedback and helped shape the research, analysis, and manuscript.

## Conflicts of Interest

The authors declare no conflicts of interest.

## Data Availability

The data that support the findings of this study are available in Google Trends at https://trends.google.com/trends/. These data were derived from the following resources available in the public domain: Google Trends, https://trends.google.com/trends/.

## References

[1] J. HE , A. Chandawarkar , and R. Kim , “Data‐Driven Insights on the Effects of COVID‐19 on Public Interest in Medical Aesthetics: Part II (Active Analysis),” Aesthetic Surgery Journal 41, no. 3 (2021): NP75–NP82, 10.1093/asj/sjaa173. 33107566 PMC7665353

[2] M. Lem , J. K. Kim , J. T. Pham , and C. J. Tang , “Effect of the COVID‐19 Pandemic on Global Interest in Plastic Surgery,” JPRAS Open 37 (2023): 63–71 20230521, 10.1016/j.jpra.2023.05.002. 37360055 PMC10200276

[3] R. Reed , “The Definition of “Cosmeceuticals”,” Journal of the Society of Cosmetic Chemists 13 (1962): 103–106.

[4] M. A. Alamer , H. Alrashed , B. M. Abuageelah , et al., “Impact of Social Media on Choosing Skin Care and Cosmetic Products Among Females in Saudi Arabia,” Cureus 15, no. 12 (2023): e49922, 10.7759/cureus.49922. 38174175 PMC10763983

[5] A. Yousaf , R. Hagen , E. Delaney , S. Davis , and Z. Zinn , “The Influence of Social Media on Acne Treatment: A Cross‐Sectional Survey,” Pediatric Dermatology 37, no. 2 (2020): 301–304, 10.1111/pde.14091. 31944359 PMC7453954

[6] V. Ranpariya , B. Chu , R. Fathy , and J. B. Lipoff , “Dermatology Without Dermatologists? Analyzing Instagram Influencers With Dermatology‐Related Hashtags,” Journal of the American Academy of Dermatology 83, no. 6 (2020): 1840–1842, 10.1016/j.jaad.2020.05.039. 32416205

[7] V. K. Ranpariya , R. Fathy , B. Chu , S. Wang , and J. B. Lipoff , “Patterns of Promotional Content by Dermatology Influencers on TikTok,” JMIR Dermatology 5, no. 1 (2022): 34935, 10.2196/34935. PMC10501523 37632857

[8] Á. Iglesias‐Puzas , A. Conde‐Taboada , B. Aranegui‐Arteaga , and E. López‐Bran , “‘Fake News’ in Dermatology. Results From an Observational, Cross‐Sectional Study,” International Journal of Dermatology 60, no. 3 (2021): 358–362, 10.1111/ijd.15254. 33095467

[9] S. M. Mueller , P. Jungo , L. Cajacob , S. Schwegler , P. Itin , and O. Brandt , “The Absence of Evidence Is Evidence of Non‐Sense: Cross‐Sectional Study on the Quality of Psoriasis‐Related Videos on YouTube and Their Reception by Health Seekers,” Journal of Medical Internet Research 21, no. 1 (2019): e11935, 10.2196/11935. 30664460 PMC6357908

[10] H. J. Feetham , H. S. Jeong , J. McKesey , H. Wickless , and H. Jacobe , “Skin Care and Cosmeceuticals: Attitudes and Trends Among Trainees and Educators,” Journal of Cosmetic Dermatology 17, no. 2 (2018): 220–226, 10.1111/jocd.12460. 29152848

[11] Google , “FAQ About Google Trends Data,”, https://support.google.com/trends/answer/4365533?hl=en.

[12] T. E. Sivesind , M. D. Szeto , W. Kim , and R. P. Dellavalle , “Google Trends in Dermatology: Scoping Review of the Literature,” JMIR Dermatology 4, no. 1 (2021): 27712, 10.2196/27712. PMC10501516 37632813

[13] J. Campbell , K. Williams , and H. Woolery‐Lloyd , “DermTok: How TikTok Is Changing the Landscape of Dermatology Patient Education,” Journal of Drugs in Dermatology 22, no. 3 (2023): 302–304, 10.36849/jdd.6676. 36877875

[14] M. R. Mansour , Y. Abushukur , and G. A. Potts , “Keratosis Pilaris on TikTok: A Cross‐Sectional Analysis of Trending Content,” JAAD International 8 (2022): 116–117, 10.1016/j.jdin.2022.06.015. 35875398 PMC9305320

[15] M. K. Patil , A. Naik , R. Chappidi , C. S. Lam , and T. N. Tran , “Dermatology in Social Media: A Cross‐Sectional Analysis of Trending Hyperhidrosis Content on TikTok,” Skin Research and Technology 30, no. 6 (2024): 13734, 10.1111/srt.13734. PMC11186035 38896018

[16] K. A. Waterton and S. R. Lipner , “Truth or Trend—Misinformation Spreading Fast on TikTok: A Cross‐Sectional Analysis of Onychomycosis Content,” Skin Appendage Disorders 9, no. 6 (2023): 444–448, 10.1159/000533319. 38058543 PMC10697755

[17] S. Kanchan and A. Gaidhane , “Social Media Role and Its Impact on Public Health: A Narrative Review,” Cureus 15, no. 1 (2023): e33737, 10.7759/cureus.33737. 36793805 PMC9925030

[18] S. Singh , “How Many People Use TikTok (2024 Statistics) Demandsage2024 [cited 2024],”, https://www.demandsage.com/tiktok‐user‐statistics/#:~:text=TikTok%20have%202.05%20billion%20registered,anticipated%20to%20reach%202.25%20billion.

[19] N. A. Charoo , “Hyperpigmentation: Looking Beyond Hydroquinone,” Journal of Cosmetic Dermatology 21, no. 10 (2022): 4133–4145, 10.1111/jocd.14746. 35020267

[20] J. S. Barbieri , J. L. Streicher , and D. Rosmarin , “Benzene in Benzoyl Peroxide‐How Worried Should We Be?,” Journal of the American Academy of Dermatology 91, no. 4 (2024): 772–773, 10.1016/j.jaad.2024.05.093. 38909658

[21] A. E. Newburger , “Cosmeceuticals: Myths and Misconceptions,” Clinics in Dermatology 27, no. 5 (2009): 446–452, 10.1016/j.clindermatol.2009.05.008. 19695475

[22] B. Tetali , F. M. Fahs , and D. Mehregan , “Popular Over‐The‐Counter Cosmeceutical Ingredients and Their Clinical Efficacy,” International Journal of Dermatology 59, no. 4 (2020): 393–405, 10.1111/ijd.14718. 31749194

[23] “A Double‐Blind, Placebo‐Controlled Evaluation of a 2% Salicylic Acid Cleanser for Improvement of Acne Vulgaris,” Journal of the American Academy of Dermatology 68, no. 4, Supplement 1 (2013): AB12, 10.1016/j.jaad.2012.12.052.

[24] M. Narda , C. Trullas , A. Brown , J. Piquero‐Casals , C. Granger , and G. Fabbrocini , “Glycolic Acid Adjusted to pH 4 Stimulates Collagen Production and Epidermal Renewal Without Affecting Levels of Proinflammatory TNF‐Alpha in Human Skin Explants,” Journal of Cosmetic Dermatology 20, no. 2 (2021): 513–521, 10.1111/jocd.13570. 32583600 PMC7891644

[25] T. Scianna , “Dermatologists Have Strongest Influence on Consumer Skin Care Decisions: MedEsthetics,” 2022, https://www.medestheticsmag.com/news/news/22314640/dermatologists‐have‐strongest‐influence‐on‐consumer‐skin‐care‐decisions.

[26] R. K. Chaudhuri and K. Bojanowski , “Bakuchiol: A Retinol‐Like Functional Compound Revealed by Gene Expression Profiling and Clinically Proven to Have Anti‐Aging Effects,” International Journal of Cosmetic Science 36, no. 3 (2014): 221–230, 10.1111/ics.12117. 24471735

[27] S. Dhaliwal , I. Rybak , S. R. Ellis , et al., “Prospective, Randomized, Double‐Blind Assessment of Topical Bakuchiol and Retinol for Facial Photoageing,” British Journal of Dermatology 180, no. 2 (2019): 289–296, 10.1111/bjd.16918. 29947134

[28] I. M. Fabian , E. S. Sinnathamby , C. J. Flanagan , et al., “Topical Hydroquinone for Hyperpigmentation: A Narrative Review,” Cureus 15, no. 11 (2023): e48840, 10.7759/cureus.48840. 38106810 PMC10723018

[29] L. Roche , E. Nic Dhonncha , and M. Murphy , “TikTok and Dermatology: Promises and Pearls,” Clinical and Experimental Dermatology 46, no. 4 (2021): 737–739, 10.1111/ced.14529. 33258146

[30] B. H. Tulbert , C. W. Snyder , and R. T. Brodell , “Readability of Patient‐Oriented Online Dermatology Resources,” Journal of Clinical and Aesthetic Dermatology 4, no. 3 (2011): 27–33. PMC3070466 21464884

